# New principle for measuring arterial blood oxygenation, enabling motion-robust remote monitoring

**DOI:** 10.1038/srep38609

**Published:** 2016-12-07

**Authors:** Mark van Gastel, Sander Stuijk, Gerard de Haan

**Affiliations:** 1 Eindhoven University of Technology, Department of Electrical Engineering, Eindhoven, The Netherlands; 2 Philips Research, Philips Innovation Group, Eindhoven, The Netherlands

## Abstract

Finger-oximeters are ubiquitously used for patient monitoring in hospitals worldwide. Recently, remote measurement of arterial blood oxygenation (SpO_2_) with a camera has been demonstrated. Both contact and remote measurements, however, require the subject to remain static for accurate SpO_2_ values. This is due to the use of the common ratio-of-ratios measurement principle that measures the relative pulsatility at different wavelengths. Since the amplitudes are small, they are easily corrupted by motion-induced variations. We introduce a new principle that allows accurate remote measurements even during significant subject motion. We demonstrate the main advantage of the principle, i.e. that the optimal signature remains the same even when the SNR of the PPG signal drops significantly due to motion or limited measurement area. The evaluation uses recordings with breath-holding events, which induce hypoxemia in healthy moving subjects. The events lead to clinically relevant SpO_2_ levels in the range 80–100%. The new principle is shown to greatly outperform current remote ratio-of-ratios based methods. The mean-absolute SpO_2_-error (MAE) is about 2 percentage-points during head movements, where the benchmark method shows a MAE of 24 percentage-points. Consequently, we claim ours to be the first method to reliably measure SpO_2_ remotely during significant subject motion.

Arterial blood oxygen saturation (SaO_2_) is defined as the fraction of oxygen-saturated hemoglobin relative to total hemoglobin in the arterial blood and as such may indicate a patient’s oxygenation status. This vital parameter is used in clinical practice for early detection of hypoxemia and is e.g. monitored during anesthesia. Nowadays, pulse oximetry is the method of choice in clinical practice and ubiquitously applied in hospitals to estimate SaO_2_ optically from the cardiac-induced absorption variations of the skin (SpO_2_). Although this *ex*-*vivo* measurement is much more comfortable for the patient compared to the earlier used *in-vitro* methods, the sensor does require contact to the skin, which may lead to skin irritation and discomfort. For the very sensitive preterm infants, the risk of not monitoring is sometimes preferred over the certain trauma caused by contact sensors^1^. This is a serious problem, for which remote monitoring, using cameras, has been recently attempted. However, the current techniques are extremely sensitive to subject movements, which limits their application to immobilized patients. This article introduces a new principle that is significantly less affected by noise, which makes it the first method that can measure SpO_2_ remotely during significant subject motion. In the remainder of this introduction we shall discuss related work, and introduce the essence of our new proposal.

Before the emerge of camera-based monitoring methods, early measurement techniques relied on analysis of arterial blood collected anaerobically from an indwelling arterial catheter or arterial puncture. These techniques do not provide immediate or continuous data. Pulse oximetry, introduced in the early 1980s by Aoyagi *et al*.^2^, is a technique for simultaneously assessment of pulse rate and oxygen saturation levels, and has rapidly gained popularity because of its low-cost, simplicity and practicality. It allows a non-invasive and continuous measurement of arterial blood oxygen saturation, SpO_2_. Pulse oximetry is based on the technique of photoplethysmography (PPG), which measures variations in blood volume optically. Applications of PPG include monitoring of pulse and respiratory rate, blood pressure and cardiac output^3^. Typically, light-emitting diodes (LEDs) with two distinct wavelengths are attached to the skin and are alternately energized. The light transmitted through or reflected from the skin is captured by a photo-detector, resulting in two PPG waveforms. Based on the different light absorption spectra for oxygenated and deoxygenated haemoglobin, a relationship can be established between the pulsatile signal strength in the two wavelength channels and the SpO_2_-level.

To provide a solution for subjects with an extremely sensitive skin, e.g. preterm infants^4^, the possibility of camera-based PPG measurement has been considered. This technique is referred to as remote PPG (rPPG). Most rPPG research focused on robust extraction of the cardiac pulse signal to measure pulse rate or derived features, e.g. heart rate variability (HRV). However, camera-based SpO_2_ measurement has also been attempted in the last few years^5^,^6^,^7^,^8^,^9^,^10^,^11^,^12^,^13^,^14^,^15^,^16^,^17^. Besides the advantage that a camera does not require direct skin contact, it has the potential to be more robust by exploiting the spatial redundancy of the camera sensor, which is not possible with the single-spot measurement of a contact sensor. However, the lower SNR of the reflected light renders rPPG methods more susceptible to noise as compared to PPG methods. Furthermore, the calibration of camera-based SpO_2_ is not trivial because of the fundamental difference between the geometries of the conventional contact source-detector and the contact-less illumination-detection; whereas the former geometry collects light that has travelled through relatively deep vasculature^18^, the latter predominantly collects light that has travelled through much shallower tissue depths over much smaller distances^19^. Currently, it is not known whether the PPG signal measured in the camera geometry stems mostly from the deeper arterioles or also (partly) from the shallow capillaries. In fact, there is some controversy on whether the PPG signal stems directly from blood volume changes at all^20^,^21^,^22^. Kamshilin *et al*.^21^ challenged the widely believed presumption that the pulsatile variations of the light absorption are mainly caused by arterial blood-volume pulsations and presented a new interpretation of remote PPG to explain their experimental observations of strongly pulsatile counter-phase PPG waveforms, which were detected as local hotspots in the amplitude and phase maps at the wrist. They proposed a new model of light interaction with biological tissue *in-vivo* in which pulse oscillations of arterial transmural pressure mechanically deform the connective-tissue components of the dermis resulting in periodical changes of both the light absorption and scattering coefficient, which suggests that it is an indirect measurement of arterial pressure variations. Moço *et al*.^22^ showed however that for the explanation of the strongly pulsatile counter-phase PPG waveforms a new physiological model is not required. They performed experiments in skin covered by opaque ink to prove that these effects find explanation in the motion pattern of the skin; ballistocardiography (BCG). A thorough understanding about the origin of the remotely measured PPG waveform is important for pulse oximetry to ensure that the cardiac-synchronous intensity variations can solely be related to arterial blood volume variations and not to other physiological factors. In their recent study, Verkruysse *et al*.^23^ showed that the fundamental difference in geometries of contact and non-contact methods does not harm the calibratibility of camera-based SpO_2_; a calibration curve determined for a population of 24 individuals was validated on 40 individuals with various skin-tones. The camera-based accuracy was found to be about ±3%, which is comparable to that of conventional transmissive probes, and therefore proves its feasibility.

The potential to measure SpO_2_ with a camera was first mentioned by Wieringa *et al*.^5^, who investigated different wavelengths for rPPG, but did not show results on oxygen saturation due to poor SNR of the PPG waveforms. Humphreys *et al*.^6^ were the first to estimate oxygen saturation with a single camera using dual-wavelength near-infrared illumination, where the PPG waveforms are obtained in transmissive mode. In ref. 7, attempts have been made to relate RGB signals to relative blood oxygen concentrations by performing Monte Carlo simulations of light transport. Scully *et al*.^8^ showed the feasibility of using the RGB camera of a mobile phone to measure oxygen saturation, although it requires the finger to be placed on the lens, similar to ref. 15 where a setup consisting of two RGB cameras is proposed with dual wavelength illumination, and ref. 24 who claim to be able to measure oxygen saturation with a smartphone camera without calibration and independent of the hardware and skin characteristics. The desire for non-contact camera-based methods was first addressed by Kong *et al*.^11^, who attached narrow-band optical filters to the cameras. They demonstrated the feasibility to estimate SpO_2_ remotely with two monochrome cameras under ambient light conditions, which has been further elaborated on by Tarassenko *et al*.^12^ by using the red and blue color channels of a single RGB camera. Another single camera approach has been presented by Shao *et al*.^16^, but instead of using ambient light illumination, a trigger-controlled dual wavelength LED-array was proposed to measure the PPG signal at different wavelengths with a monochromatic camera. Recently, efforts have been made to minimize the effects of noise artifacts, which have a large influence on the accuracy of the described methods. Bal *et al*.^14^ proposed using a skin detector to only select skin pixels from the detected face region, whereas Guazzi *et al*.^13^ exploited the spatial redundancy of the camera by pruning distorted regions based on signal quality and phase information. All aforementioned methods are based on the principles of conventional pulse oximetry, which determine the blood oxygenation levels from parameters directly extracted from the PPG waveforms measured at two wavelengths. In other words, the current methods measure relative pulsatility at different wavelengths. This makes them susceptible to noise and hence their robustness is limited. The accuracy is further influenced by the sensitivity of rPPG methods to motion. None of the aforementioned methods has addressed this challenge limiting their use for clinical practise.

We introduce a new principle that allows accurate measurement even during significant subject motion. It is based on a recent technique^17^ exploiting an a priori signature of relative pulsatile amplitudes at different wavelengths to extract the best quality pulse-signal from noisy data. Basically, we invert the principle of robust pulse-extraction by searching for the signature that produces the best pulse quality. This optimal signature then can be mapped to an SpO_2_-reading. The large asset of our method compared to all current methods is that it does not require clean PPG signals for accurate measurement, instead it utilizes the fact that noise and motion artifacts affect the overall quality of the pulse signal, but not the optimal wavelength combination which minimizes distortions. This optimal combination is shown to remain stable, even when the signals are very noisy; results remain accurate with noise levels that can be 3 orders of magnitude higher than what the ratio-of-ratios method, used in conventional pulse oximetry, allows. Additionally, the spatial redundancy of the camera is exploited to further reduce the influence of artifacts on the measurement by using multi-site measurements. This improvement is shown to be exclusively applicable to the new principle, since the increased noise in the smaller sub-regions prohibit current ratio-of-ratios methods to profit from such multi-site measurements. To our knowledge, this is the first non-contact camera-based method to measure blood oxygenation levels in the presence of motion and noise artifacts.

## Results

The experimental results can be categorized into two sections. The first section consists of quantitative noise sensitivity results. A 4 minutes recording without subject motion containing a rapid desaturation event has been selected, where multiplicative random noise with different noise levels has been added to the recording to verify the robustness of the method, both with and without exploiting multi-site measurements. The second section presents the results from a self-created dataset of healthy subjects with various skin-tones, which perform head movements to verify motion robustness. The experimental setup of all recordings consists of three identical monochrome cameras, type Manta of Allied Vision Technologies GmbH, which capture the scene simultaneously at a frame rate of 15 FPS, with a resolution of 968 × 728 pixels and with 8 bits depth. The cameras have 25 mm lenses with different optical filters mounted to them, each capturing a specific part of the light spectrum. For our benchmark dataset optical filters with a center-wavelengths of 760, 800 and 840 nm are used. The reasoning for this wavelength selection is twofold: (1) the clinical desire to measure SpO_2_ in full darkness, e.g. during sleep in a hospital setting, and (2) the wavelengths are spectrally sufficiently spaced to provide contrast necessary for robustness, while remaining within the spectral sensitivity of the camera sensor. The corresponding pulse vectors for this wavelength combination are: 
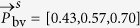
 and 
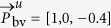
. More details about these vectors and how they can be used to measure SpO_2_ can be found in the Methods section. Since the three monochrome cameras have slightly different viewpoints because of their physical spacing, the frames are aligned using an affine transformation to ensure that the pixel locations are coinciding for the different channels. For reference, a finger pulse-oximeter has been attached to the index finger of the subject. This data is synchronized with the camera frames. The performance of the algorithms is evaluated with three different metrics: (1) Mean Absolute Error (MAE), (2) Root Mean Squared Error (RMSE) and (3) standard deviation (STD):


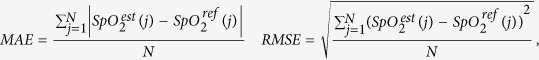


where *N* indicates the number of evaluated samples. Additionally, correlation and Bland-Altman analysis have been performed. Our method, entitled adaptive PBV (APBV), is benchmarked against a ratio-of-ratios-based (RR) algorithm, in line with the earlier proposed camera-based methods. This benchmark algorithm is extensively described in the Methods section, and is calculated with the 760 and 840 nm PPG waveforms. The SpO_2_ values have been estimated on time-windows with a duration of 10 seconds, with a step-size of 1 second.

### Noise sensitivity analysis

As indicated in the introduction, motion and noise artifacts have a large influence on the performance of conventional pulse oximeters. Earlier proposed camera-based methods suffer from the same limitation. To verify the sensitivity of our method to these artifacts, a recording with a duration of 4 minutes has been selected which includes breath-holding after 1 minute to induce a hypoxemic event, resulting in a dip in oxygen saturation of more than 10 percentage-points (PP). The recording was made under incandescent lighting conditions, providing homogeneous illumination of the skin area. Incandescent light was used because of its continuous emission spectrum for both visible and infrared wavelengths. However, we only use invisible portions of the emitted spectrum, and consequently the light source can be replaced with nearly invisible LEDs. By evaluating the performance of our method for different noise levels, the accuracy of our method can be measured and compared with the benchmark algorithm. The noise-adding process is defined as:


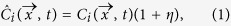


where 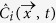 identifies the value of the pixel at location 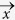 and time *t* for wavelength *i*, and *η* indicates the zero-mean random Gaussian noise term added to the spatial average of each sub-region at time *t*. The resulting multiplicative noise is similar to the distortions caused by intensity variations, typically seen during motion. An advantage of using a camera compared to a contact probe is that it allows multi-site measurements.

**Figure 1:**
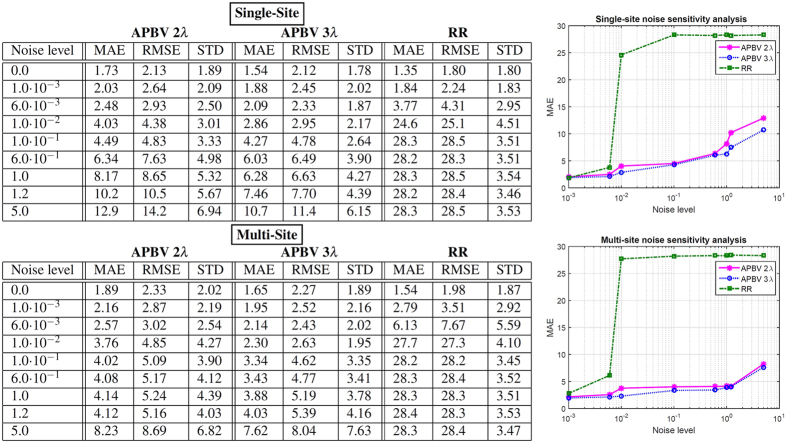
Results of the noise sensitivity analysis on a recording without motion including a hypoxemic event. We compare the performance of our method, both with two and three wavelengths, with the benchmark RR method for different noise levels. The top row contains the results using a single-site measurement, the bottom row that of using multi-site measurements.

Regions with a distorted signal within the selected Region of Interest (RoI) which pollute the measurement can be pruned when exploiting this spatial redundancy. To individually assess the gain in performance of our principle and the contribution of using multi-site measurements, we first perform the analysis on the single-site measurement of the entire RoI, which is subsequently repeated using multi-site measurements, where the RoI is divided into equally-sized sub-regions. The details how we exploit the spatial redundancy are described in the Methods section. Figure 1 contains the statistical results of the analysis.

The results show that the APBV method profits from multi-site measurements, in contrast to the RR method, which even shows a decrease in performance. This can be explained by the reduced SNR of the sub-regions compared to the SNR of the entire ROI. Our method is not much affected by noise in the individual PPG waveforms since the algorithm searches for the signature that provides the best SNR, even though it may be quite low. However, the performance of the RR method is highly affected by the signal quality. Averaging over more skin pixels improves signal quality and hence improves the accuracy of the RR method.

**Figure 2:**
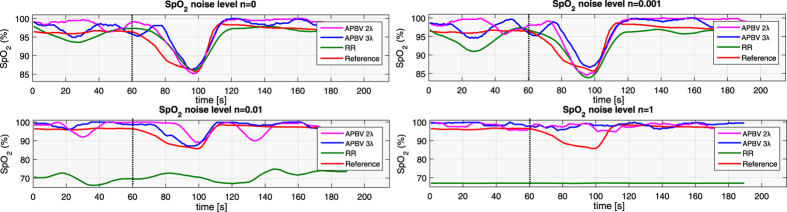
Noise sensitivity results after adding multiplicative Gaussian distributed random noise to a stationary sequence with a breath-holding event, whose start is indicated with the dotted line. It can be observed that the proposed method is much less sensitive to noise compared to the standard ratio-of-ratios (RR) method, and three wavelengths yield better robustness compared to two.

**Figure 3:**
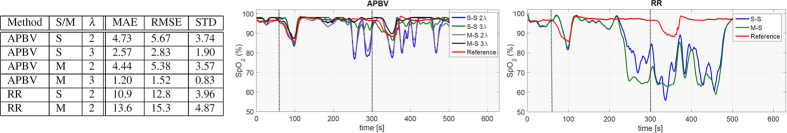
Results on a 9 minutes motion sequence including two hypoxemic events (indicated with dotted lines). We compare the performance of our method, both with two and three wavelengths (*λ*), with the benchmark RR method. Furthermore, we investigate the gain in performance when using multi-site measurements (M-S) compared to a single-site measurement (S-S).

**Figure 4:**
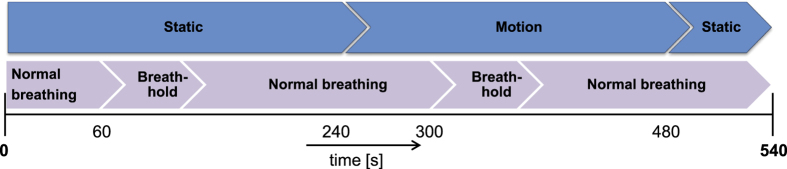
Overview of the protocol for the dataset of motion robust oxygen saturation measurements.

Our method requires at least two wavelengths. However, when using two instead of three wavelengths as used in the original formulation^17^, one loses dimensionality to suppress distortions, which will negatively impact the performance of our method. To verify this hypothesis, we compared the performance of our method for both two and three wavelengths, where the two wavelengths are similar to the ones selected for the RR method. The results show that the RR-based benchmark algorithm is able to accurately estimate SpO_2_ for noise levels up to 0.006 when using a single-site measurement, whereas the APBV method is capable to estimate SpO_2_ for noise levels higher than 1, which indicates the large improvement in robustness of our method. The clinically acceptable accuracy criterion is specified in the International Standard for pulse-oximeter manufacture ISO 80601–2–61–2011, which requires an accuracy of ≤4% error in the range 70–100% SpO_2_^25^. Comparing the results obtained with two and three wavelengths, confirms our hypothesis that robustness decreases when reducing the number of wavelengths. Figure 2 provides a visual comparison for different noise levels when using multi-site measurements for the APBV method and an average-site measurement for the benchmark RR method, as these combinations provide the best results. These results are obtained from a simulation of an ideal case, where motion causes intensity-variations only. We expect the performance-drop to be more severe in the real-life case where the noise may have a different character. To verify this hypothesis, we repeated the previous analysis on a motion sequence. The results of this analysis are presented in Fig. 3 and the protocol used is described in Fig. 4. Real-life artifacts, like remaining parallax, tracking errors, specular reflection, may additionally lead to more harmful color-distortions. The results indeed show that the differences between the different evaluated variants have increased, especially those between two and three wavelengths. Based on these results, we decided to create our dataset with three wavelengths.

### Breath-holding events with motion

The results from the noise analysis show that our proposed method outperforms the benchmark algorithm in terms of robustness to multiplicative, random noise. Additionally, we found from analysis on a motion sequence that our method profits from multi-site measurements. To verify if this also holds on a broader population, we created a dataset where subjects were asked to perform continuous quasi-periodic head movements, similar to the protocol of the previous Section. A total number of 14 recordings were made of 4 healthy, non-smoking subjects with different skin-types in the range II-V of the Fitzpatrick scale^26^. We had to exclude 2 recordings from the dataset because one suffered from frame drops during acquisition and for one other our motion-tracker could not deal with the vigorous motion. An overview of the 9 minutes protocol is visualized in Fig. 4. The subjects were asked to have their face focussed towards the cameras in front of them. During the first four minutes of the recording, the subjects were asked to keep their head stationary and hold their breath as long as possible starting one minute after the beginning of the test. Between the fifth and eighth minute, a second breath-holding event was timed similar to the first event, however the subjects were now asked to move their head for four minutes with a combination of translation and rotation movements. The last minute of the 9 minutes protocol was stationary again with normal breathing. The duration of the entire dataset is 108 minutes with SpO_2_ values ranging between 80.2 and 100%.

**Figure 5:**
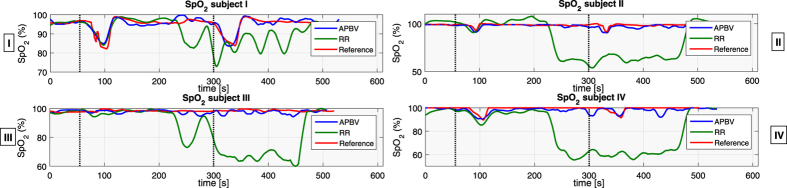
Performance results of the four (I–IV) subjects present in our dataset, where the dotted lines indicate the beginning of the breath-holding events. It can be observed that the RR-based method achieves satisfactory results in the first static part of the recordings, but completely fails in the presence of motion artifacts. The proposed APBV method is capable to estimate SpO_2_ during motion, although a decrease in accuracy can be identified compared to the static part.

In Fig. 5, the results of all four subjects, indicated I–IV, are displayed visually. Based on the noise sensitivity results, the APBV method is evaluated using multi-site measurements, whereas the RR method uses single-site measurements. It can be observed that both the benchmark RR method and our APBV method are capable of estimating SpO_2_ during the static part of the sequence, when there is no head motion. During the head movements in the second part of the sequence, the RR method completely fails, whereas the APBV method is still able to estimate SpO_2_, although accuracy decreased compared to the static part. The statistical results are presented in Table 1. To quantify the difficulty of the sequences, both the pulse amplitude and the average noise amplitude are calculated for each subject, which are defined as the ratio of the pulsatile AC component and the stationary DC component. Here the AC component is determined by calculating the standard deviation of the concatenated spatial mean values of the 800 nm wavelength after filtering out the pulse frequency and its harmonics, and the DC component is determined by the first component of the Fourier transform. Besides amplitudes, also the range of pulse rates and motion frequencies present in the dataset are added for each subject.

**Figure 6:**
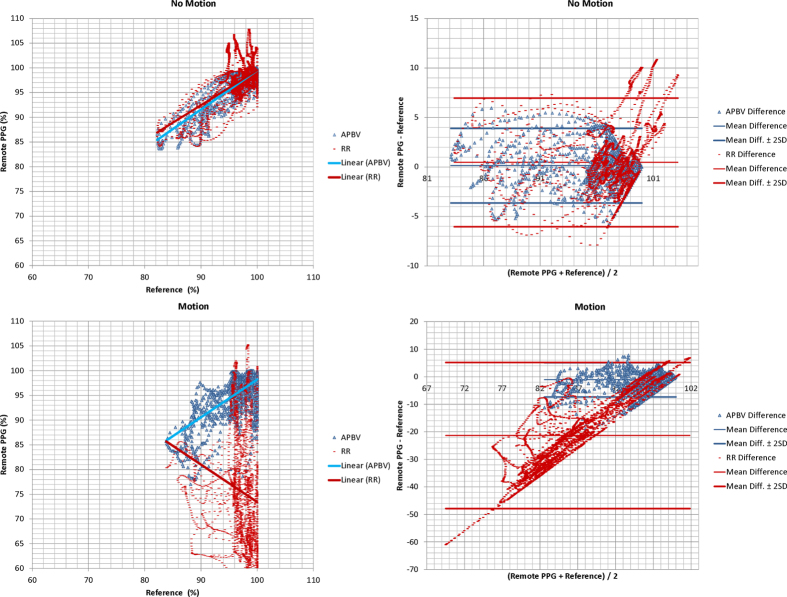
Correlation plots (left) and Bland-Altman analysis (right) of our method compared to the benchmark method, which are split in static and motion sequences. The red and blue lines in the correlation plots indicate the linear fit.

During the static part of the sequences, the estimated SpO_2_ of our APBV method is within the clinically acceptable accuracy of ≤4% for 96.6% of the time, compared to 87.9% for the benchmark RR method. The inaccuracies in the static parts are mainly caused by physiological response differences between head and finger location, which will be further discussed in the next Section. In the challenging motion part of the sequences, the APBV method is clinically accurate according to the ISO standard^25^ for 86.1% of the time, in contrast to the benchmark method which is clinically accurate for only 13.1% of the time. Correlation plots and Bland-Altman analysis are displayed in Fig. 6, whereby the 95% limits of agreements are set at 2*σ*. We split our analysis into two parts: static and motion, to distinguish between the performance of the methods for both scenarios.

- **Static**: The average MAE of the APBV method is 0.90 PP versus 2.51 PP for the RR method. The slope of the linear fit *B* is 0.93 for the APBV method with a Pearson correlation coefficient *r* of 0.94, compared to *B* = 0.91 and *r* = 0.92 for the RR method. Both methods have a small positive bias: 0.14 and 0.46, respectively.
- **Motion**: The average MAE of the APBV method is 2.03 PP versus 24.2 PP for the RR method. The slope of the linear fit *B* is 0.82 for the APBV method with a Pearson correlation coefficient *r* of 0.83, compared to *B* = −0.76 and *r* = −0.78 for the RR method. The APBV has a small negative bias, −1.13, whereas the RR method has a large negative bias, −21.4.

Overall, the average MAE on the complete dataset is 2.03 PP for the APBV method, compared to 24.2 PP for the benchmark method. This difference is mainly caused by the sensitivity to motion artifacts of the benchmark algorithm, and emphasizes the improvement of our proposed method to cope with these distortions.

## Discussion

The reference blood oxygenation values for our dataset are obtained with a pulse-oximeter attached to the index finger of the left hand. It is well known that finger-oximetry readings lag the patient’s physiologic state; signal averaging of 4 to 20 seconds is typical of most monitors^27^. A delay because of sensor anatomic location and abnormal cardiac performance compound the lag relative to central SaO_2_. Forehead and ear probes are closer to the heart and therefore respond more quickly than distal extremity probes; an average delay of 15 seconds between ear and finger has been measured^28^. The response difference compared to central SaO_2_ is also compounded by hypoxemia and slower peripheral circulation such as low cardiac output states. As such, forehead reflectance probes are often preferred in critically ill patients. All of these response delays become clinically more important during rapid desaturation, such as those present in our dataset. Since we estimate SpO_2_ from the facial skin, an ear probe would be the optimal solution to minimize the response time without occluding skin pixels. However, since the subjects perform head movements, this probe location suffers from motion artifacts resulting in erroneous measurements. A finger can be easily isolated from these head movements and was therefore selected as best alternative location. This measurement location as reference imposes two assumptions: (1) a fixed response delay between head and finger, and (2) a similar response at both measurement sites, which holds in steady-state but not during hypoxemia, e.g. during breath-holding events^29^. Therefore the comparison of saturation values between our method and the reference may not be accurate during these events, although a dip in the SpO_2_-curve is expected to occur at some, not too distant, point in time.

**Figure 7:**
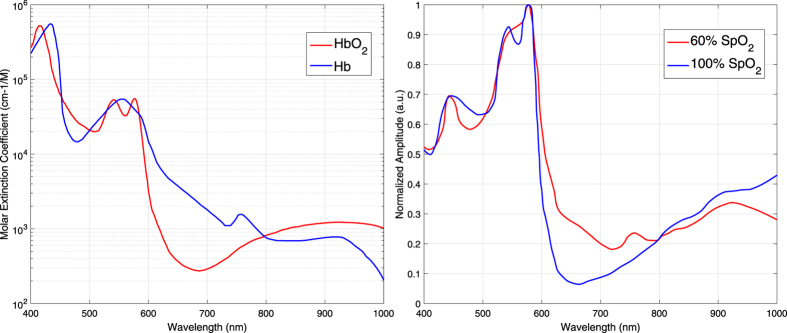
Left: Optical extinction spectra of oxygenated haemoglobin (HbO_2_) and deoxygenated haemoglobin (Hb)^41^. Right: The relative PPG amplitude spectra for 60% and 100% SpO_2_.

**Figure 8:**
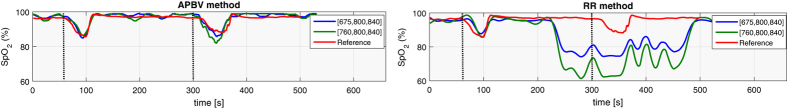
Visualization of the comparative results for the wavelength combinations [675,800,840] and [760,800,840] nm on a sequence with motion. The dotted lines indicate the beginning of the breath-holding events.

As can be observed from Fig. 7, when SpO_2_ decreases, the pulse amplitude at 760 nm increases whereas the amplitude at 840 nm decreases. Since the amplitude at 800 nm does not change as it is close to the isosbestic point of (oxy-)haemoglobin, the differences in amplitude decrease between the three channels. A consequence is that motion robustness of the PBV method decreases at lower blood oxygenation values. Assuming uniform, homogeneous illumination of the skin, motion will result in intensity variations which are equal in all three channels. Similar to the definition of 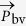, these variations can be characterized with the ‘motion’ vector [0.58, 0.58, 0.58]. Since the pulse ‘signature’ 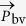 at normal blood saturation levels is different from this motion signature, the PBV method is able to suppress these motion distortions. However, when the pulse and motion signatures are becoming more similar, as it occurs during de-saturation, it gets more difficult to discriminate between them, resulting in a pulse signal with lower SNR. The selection of wavelengths has a large influence on the robustness of the method. Motion robustness can be improved by maximizing the angle between the pulse and motion vector, as has been investigated in our previous study^30^. Our wavelength selection is mainly motivated by the clinical desire to measure SpO_2_ in full darkness. However, when full darkness is not a strict criterion, it is preferred to increase the spectral spacing between the wavelengths to increase contrast. To illustrate this point, we added a fourth wavelength with a center-wavelength of 675 nm to the experimental setup. To verify the gain in performance, another recording with the described 9 minutes protocol was made, where we compared the performance for two combinations of wavelengths: [675,800,840] and [760,800,840] nm. The results are displayed in Fig. 8, and confirm the improved robustness of our method with the 675 nm wavelength. Because also the RR method profits from an increased contrast, the comparative results of this method are added to the figure. Similarly, some gain can be expected choosing the longest wavelength even longer. There is a limitation here, in that the camera-sensitivity drops rapidly for longer wavelengths, while also water-absorption may start to play a role.

**Figure 9:**
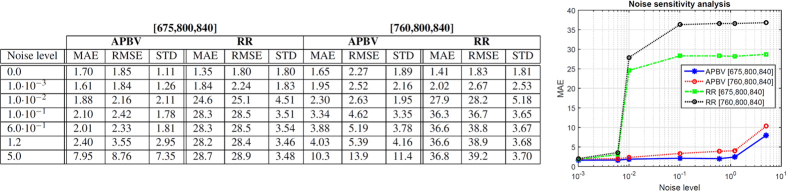
Comparative results of the noise sensitivity analysis for the wavelength combinations [675,800,840] and [760,800,840] nm.

Statistical results are presented in Table 2. As expected the performance does not change considerably during the static part of the recording; an average error of 1.17 versus 1.21 PP. In the challenging, motion part of the sequence however, a noticeable gain in accuracy can be recognized; an average error of 1.40 compared to 1.98 PP when using 675 nm. Since the motion distortions are difficult to quantify in sequences where subjects are asked to move their head continuously, we performed a noise sensitivity analysis similar to the one described in the Results section. The results of this analysis are presented in Fig. 9, and confirm our previous results that clinically acceptable accuracy can be achieved at higher noise levels when the shortest wavelengths is moved towards visible red. Please note that this change of wavelength improves motion robustness at normal oxygen saturation levels, but is not a solution to the reduced robustness at low saturation levels since the angle between the pulse and motion vector still decreases for decreasing SpO_2_ levels.

A common problem in pulse-oximetry is the decreased accuracy for individuals with a dark skin, especially the overestimation of SpO_2_ during de-saturation^31^. Because of the higher melanin content of their skin, more light is absorbed, resulting in reflected light with a lower pulsatile amplitude compared to skin with a lower pigmentation level. Skin reflectance is rather uniform in the infrared part of the light spectrum, as measured by ref. 32. Consequently, whereas both the AC and DC components decrease for increasing melanin content, the ratio of both remains stable, resulting in a stable signature-vector 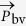 over the entire range of skin pigmentation levels, as verified in our large scale study^30^. As a result, the calibration coefficients, i.e. the values of the static and update signature-vectors of our method, are expected to be independent of the skin pigmentation level. Our dataset contains one subject, subject II, with Fitzpatrick skin-type V to challenge this hypothesis. The result of this sequence is visualized in Fig. 5. It can be observed that there is indeed no decrease in accuracy visible for our method, especially during the two de-saturation events, whereas the ratio-of-ratios based method overestimates SpO_2_ in the static part of the sequence, which is likely caused by the reduced SNR of the PPG waveforms. It should however be noted that in contrast to our previous large scale study with 40 subjects, this ‘proof of concept’ dataset only includes one dark-skinned subject with SpO_2_ values in a rather narrow range.

The values of the PBV signature-vector for an arbitrary wavelength combination can be calculated by Equation 16. In our method, we assumed the PPG amplitude spectrum to be the only time-varying factor in this expression. However, it may occur that the illumination spectrum *I(λ*) varies over time, e.g. due to ambient light interference, or the simulated light spectrum deviates from the actual light spectrum. From Equation 16, it can be observed that this will affect the values of the PBV vector, and jeopardizes the accuracy and calibratibility of our method, but also all existing methods will suffer from this problem. However, in most clinical environments illumination is strictly controlled, and chromaticity changes of the light source are not likely to occur. Moreover, the problem can be prevented with narrow-bandwidth optical filters. This makes results virtually independent of the illumination spectrum.

Our method focusses on application in near-infrared. However, our method could also be applied in visible light, in the wavelength range [400–700] nm. As can be observed from Fig. 7, it are mainly the red wavelengths which are affected by changes in SpO_2_, whereas the shorter wavelengths only vary slightly. The PBV method has originally been developed for applications in visible light using an RGB camera. By only adjusting the values of the PBV vectors, one could apply our method in visible light to arrive at a more motion-robust version of Guazzi *et al*.^13^. A critical remark should however be made about the choice for the blue and red color channels of their RR-based method. Because of the shallow penetration depth of blue, it suffers much more from specular reflections compared to the other two color channels. Since SpO_2_ is determined by measuring relative pulsatilities of arterial blood at different wavelengths, it is likely that the reported measurements are corrupted by these artifacts and hence jeopardizes the calibratibility of the method.

A potential risk for measurements in visible light is the temperature dependency, which makes calibration much more difficult. Since green and particularly blue wavelengths have much shallower skin penetration depths compared to red and near-infrared, the reflected light is mostly determined by blood-absorption in the capillaries close to the surface, and much less by the blood-volume variations in the arterioles. Since capillaries are closer to the skin surface, environmental temperature will have a large influence on these, but much less on the arterioles, which are located in the deeper layers of the skin and will therefore have a temperature close or equal to the body temperature. It has been observed that the relative PPG-amplitude is positively correlated with skin-temperature^33^. At low ambient temperatures, the viscosity of blood increases, which together with sympathetic-mediated vasoconstriction will decrease blood flow in the cooled skin-region. This risk of environment temperature dependence may however not be an issue, because of the strictly controlled environmental temperature in most clinical settings.

In summary, our work builds on the calibratibility proof of camera-based SpO_2_ measurement in near-infrared, and uses the same signal acquisition as in ref. 23. Our new principle for SpO_2_-estimation, however, radically changes the signal processing to arrive at a significant motion robustness for the contact-less scenario. We first proof our new measurement principle to be an improvement over RR on noisy data. Next, we show the unique characteristic of the new principle that it can operate on noisy data, enabling us to add multi-site measurements on small regions which are problematic for RR due to lower SNR, and combined it with a relatively simple motion-tracker to show that this results in a much increased performance. We would like to emphasize that the obtained results will depend on the quality of the tracker, and the parameter choice of the multi-site measurement. We did not attempt to fully optimize these system aspects, and also cannot claim clinical validity of our proposal. The method has been validated on sequences with motion frequencies both inside and outside the heart rate frequency band. We recognize, however, that it is possible to imagine a scenario, e.g. if continuous periodic head movements with a constant frequency similar to the pulse rate would occur, in which our method will likely be inaccurate. This should be expected, since to determine oxygenation levels we estimate an SNR which will be incorrect if the distortion frequency and the pulse-frequency coincide. Although such scenario might occur, e.g. in a fitness setting where exercise causes periodic motion, we expect it to be unlikely in a clinical setting. Since an SpO_2_ measurement seems less relevant for fitness, we did not include this scenario in our dataset.

Our results have been obtained on a dataset recorded in a controlled environment on healthy subjects to proof the concept. For future work, we are planning to perform a clinical validation of our method in a hospital setting on patients suffering from obstructive sleep apnea (OSA). These patients can have very low and rapidly varying oxygenation values in combination with body movements, which are not easy to simulate by healthy subjects. In current practise, OSA patients are monitored with contact sensors during sleep, causing stress and discomfort. Our method could be a valuable alternative to mitigate these side-effects while simultaneously eliminating the use of disposables.

## Methods

Conventional pulse oximetry estimates blood oxygen saturation levels by extracting features from multiple PPG waveforms measured at different wavelengths. The earlier proposed contact-less camera-based methods are based on a similar principle, e.g. ref. 13, and will therefore be used as benchmark for our method. This section is organized as follows: (1) we will first concisely derive the principles of conventional pulse oximetry and present the adaptations made for our benchmark algorithm to improve robustness to noise and motion artifacts, (2) explain the robust pulse extraction method, and (3) present how we exploit this method to measure SpO_2_ robustly.

### Ratio-of-ratios method

Arterial blood oxygen saturation is defined as the ratio of oxyhaemoglobin (HbO_2_) to total haemoglobin; the iron-containing protein which serves as the oxygen-carrier in blood:


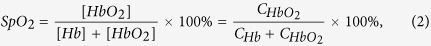


where 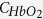, *C*_*Hb*_ are the concentrations of oxyhaemoglobin and deoxyhaemoglobin, respectively. The theory of conventional pulse oximetry has been described in several publications^2^,^34^,^35^ and is referred to as the “ratio-of-ratios” method. Let us now derive this method which is used as benchmark and will later be used to explain our method. Pulse oximetry measures SpO_2_ non-invasively and continuously by applying spectroscopic techniques and the Beer-Lambert law, which describes the transmission of light through a material as a function of incident light intensity (*I*_0_), extinction coefficient (*ε(λ*)), path length of the light (*l*), and concentration of the substance (*C*):


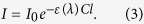


Figure 7 shows the electromagnetic radiation extinction spectra for oxygenated and deoxygenated haemoglobin in the visible and near-infrared regions of the light spectrum. As can be observed, deoxygenated haemoglobin absorbs more red light in contrast to oxygenated haemoglobin, which absorbs more infrared light, for *λ* > 800 nm. By comparing the optical extinction at these two regions of the light spectrum, a pulse oximeter can distinguish between the two haemoglobin species. Typical wavelengths of pulse oximeters are 660 and 940 nm, and are selected based on locations in the spectra where relatively large extinction coefficient differences between the two haemoglobin species are present. When the light is emitted through a peripheral site, there are many tissues with different path lengths, concentrations and extinction coefficients contributing to the light attenuation. The Beer-Lambert law, Equation 3, can be split into three components: attenuation due to arterial Hb, attenuation due to arterial HbO_2_, and attenuation due to other tissues. Their contributions to the overall light extinction are assumed to be additive:


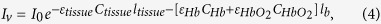


where *l*_*b*_ is the path length through the arterial blood, and *l*_*tissue*_ is the path length through other tissues. This equation applies between pulses of arterial blood, i.e., at the valley of the PPG waveform (*I*_*v*_).

Pulse oximetry uses the pulsatile nature of arterial blood to isolate the Hb and HbO_2_ terms. When an arterial blood pulse enters the peripheral site, the arteries dilate and the path length through the arterial blood changes slightly (Δ*l*). For the light attenuation at the peak of the arterial pulse (*I*_*p*_), a second light absorption equation can be set up:


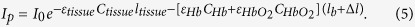


The Hb and HbO_2_ terms can now by isolated by dividing Equation 5 by Equation 4:


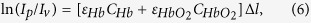


where by the effects of other tissues and the power of the incident light are cancelled out. This expression depends on the path length difference Δ*l*, which is unknown. A new equation can be established by changing the incident wavelength. By examining Equation 6 for two different wavelengths, *λ*_1_ and *λ*_2_, and taking the ratio, its dependence on Δ*l* can be eliminated assuming an equal path length through the arterial blood for both wavelengths:


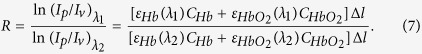


By using Equation 2, Equation 7 can be expressed as:


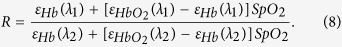


In this form the ratio *R* is not a function of the optical path length and can be derived from the arterial oxygen saturation instead of the concentration of the haemoglobins in the blood. Finally, Equation 8 can be rewritten in a form where *SpO*_2_ is a function of the ratio *R*:


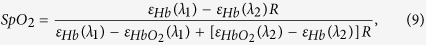


where the values *ε* at a specific wavelength can be read from Fig. 7. In practice, however, the values of R are often mapped to SpO_2_ values using empirical calibration since manufacturing tolerances in the illumination cause the oxygenation value to vary, and additionally Beer-Lambert law does not take into account the wavelength-dependent effect of scattering:


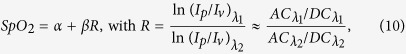


where the calibration coefficients *α* and *β* are determined by regression or selected from a look-up table based on calibration curves, and AC/DC indicate the pulsatile and non-pulsatile components of the PPG waveform, respectively. This approximation of *R* derived from the original formulation 7 holds, since the differences between peak and trough values in the signals are typically very small. Essentially, ratios-of-ratios measures relative pulsatility at two different wavelengths and maps the ratio of both to an SpO_2_ value.

Despite the straightforward relation between *R* and *SpO*_2_, there is no unambiguous method for the estimation of the AC and DC components of the PPG waveforms. Typically, the AC component is determined by calculating the peak-to-valley amplitudes of the PPG waveform, indicating the intensity difference between systolic and diastolic phase of the cardiac cycle, whereas the DC component is typically determined as the average of these peak and valley values. This approach performs satisfactorily when the PPG waveforms are not distorted by noise and motion artifacts. However, the signal-to-noise ratio (SNR) of camera-based PPG signals is much lower compared to those obtained by contact PPG. Therefore, we seek to minimize the distortions which pollute the measurement similar to the proposed camera-based methods described in the introduction Section, and use this method as benchmark algorithm for our analysis. First, the blind source separation technique Independent Component Analysis (ICA) is performed on the temporally-normalized traces of all three color channels. Similar to ref. 36, the component with the largest energy peak in the heart rate band of the frequency spectrum is selected as pulse signal, 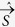, and the corresponding energy peak as pulse rate:


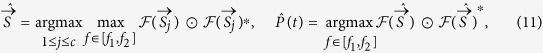


where *c* indicates the number of independent components, 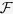 is the Fourier transform, and *f*_1_, *f*_2_ are the minimum and maximum plausible pulse rates respectively, typically set at [0.8,4] Hz for adults. The estimated pulse rate, 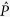, is used for the design of the narrow-band adaptive bandpass filter, which eliminates all frequencies not associated with the cardiac pulse signal. The AC components are consequently estimated by calculating the median of the time-domain detected peak-valleys values of the filtered signals within the time-window, whereas the DC components are estimated by taking the median of the low-pass filtered signals, with a cut-off frequency of 0.05 Hz. From the original three wavelengths, the two wavelengths with the largest contrast, e.g. the wavelengths which have the largest absorbance differences between Hb and HbO_2_, are typically selected for the calculation of SpO_2_. The calibration coefficients *α* and *β* are determined by performing linear regression on all static sequences from all subjects, and are therefore not patient specific. We applied the same calibrations coefficients on all sequences and subjects based on the findings of the recent calibration study of Verkruysse *et al*.^23^. For the multi-site measurements, the RR method uses features similar to the ones presented in the Framework section to prune distorted sub-regions. However, instead of calculating an average pulse-signal from the non-pruned sub-regions, the AC and DC components are determined by calculating the mean of the AC and DC components from the non-pruned sub-regions.

### PBV method

Current research in the field of remote PPG mainly focusses on extracting the cardiac pulse signal in the presence of motion and noise artifacts. Recently, de Haan *et al*.^17^ presented a method, the “PBV-method”, which uses the unique ‘signature’ of the blood volume pulse signal. An interesting property of this method is that it utilizes the different relative pulsatile amplitudes in the color channels to differentiate between intensity variations induced by blood volume changes and variations which are not related to these. Since SpO_2_ affects the pulsatile amplitudes of the color channels, this can be exploited to measure blood oxygenation values as will be shown later. We will first summarize the PBV method, and consequently explain how this method can be adapted to measure SpO_2_ in the next Section.

De Haan *et al*. showed that the minute optical absorption changes caused by blood volume variations in the skin occur along a very specific vector in a normalized RGB-space. This unique blood volume signature enables robust rPPG pulse extraction that minimizes the contribution to the pulse-signal of color variations with other signatures. Compared to the motion robust chrominance-based pulse-extraction method by de Haan *et al*.^37^, no assumptions about the distortion signals have to be made. Instead, the known relative pulsatile amplitudes 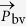 in the mean-centered normalized color channels are employed to discriminate between the pulse-signal and distortions.

We assume that the pulse-signal 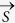 can be constructed as a linear combination of the three normalized color channels:


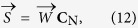


where 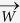, dimensions 1 × 3, is the weighting matrix with 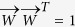, and **C**_N_ has dimensions 3 × N. where N indicates the number of samples in the time-window.

Since the relative pulsatile amplitudes in the color channels of the camera are known based on physiology and optics, the aim is to find the weights, 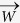, that construct the pulse-signal 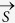, for which the correlation with the normalized color channels equals 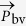:


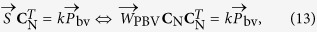


and therefore the weights 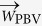 can be calculated using:


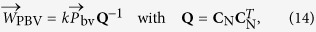


where scalar *k* is chosen to ensure that 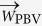 is normalized in ℓ^2^-norm sense. To employ the PBV-method and extract the cardiac pulse-signal, the relative pulsatile amplitudes of the channels, compiled in the normalized blood volume pulse vector 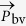, have to be known.

Let us summarize the prediction of the pulse vector from physiology and optics following^17^. The *relative*, AC/DC, PPG amplitude as function of the wavelength *λ* has been *modeled* by Hülsbusch^38^. Corral^39^ *measured* the *absolute*, i.e. AC, PPG spectrum using a tungsten-halogen lamp as illumination, which emits radiation in both the visible and NIR section of the light spectrum. The relative PPG can be related to the absolute PPG by:


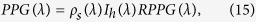


since the light-source and the skin determine the baseline component of the absolute PPG spectrum. Here *ρ*_*s*_(*λ*) and ***I***_*h*_(*λ*) represent the skin reflectance spectrum and the emission spectrum of the tungsten-halogen illumination, respectively. The relative pulsatile amplitudes in the three channels of a camera, described by the blood volume pulse vector 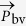, is given by:


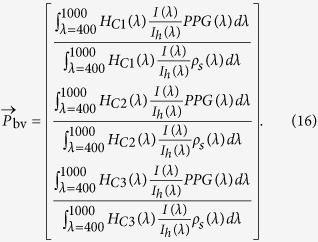


**Figure 10:**
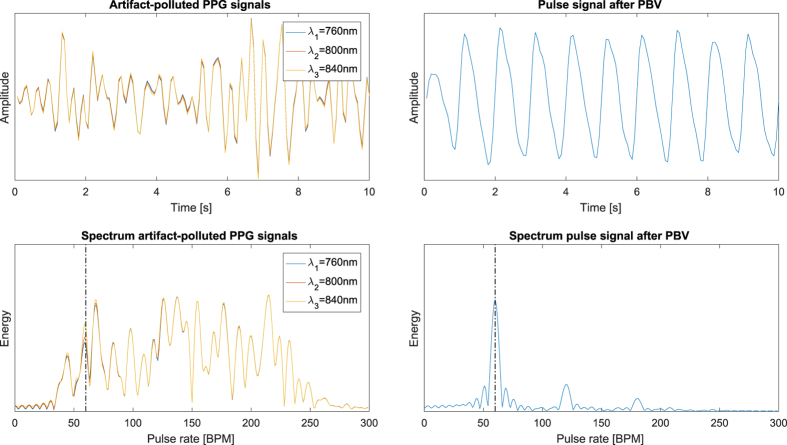
Illustration of the ability of the PBV method to suppress non-cardiac related distortions present in the PPG signals. (Left) artifact-polluted PPG signals with corresponding spectra where the distortions are dominant in energy compared to the pulse signal. (Right) the resulting pulse signal and corresponding spectrum after applying PBV. The black dash-dotted lines indicate the pulse rate.

Here *H*_*C*1,*C*2,*C*3_ are the responses of the three channels, respectively, and *I(λ*) is the spectrum of the illuminant. De Haan *et al*. demonstrated the robustness of the method for fitness applications in visible light with a regular RGB camera, which has later been extended for applications in infrared by Van Gastel *et al*.^30^. An example to illustrate the ability of the PBV method to suppress distortions present in the PPG signals is displayed in Fig. 10.

### Adaptive PBV method

**Figure 11:**
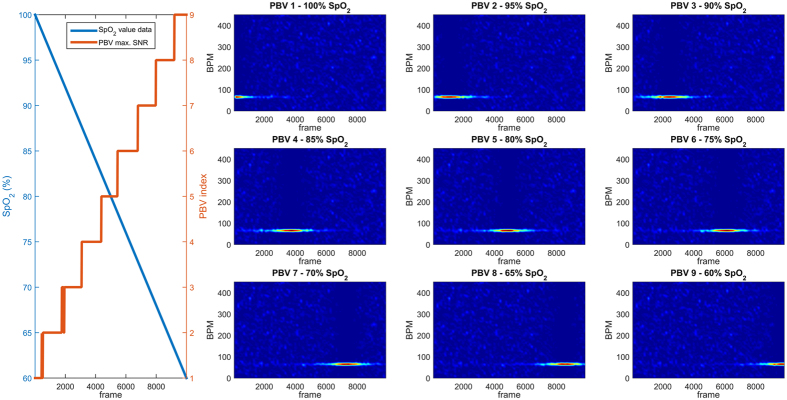
Illustrative example to demonstrate the principle of the adaptive PBV method. Synthetic data with added noise (A_pulse_ = 2 · 10^−3^, A_noise_ = 1 · 10^−2^) is generated to simulate a slow, linear, desaturation event with oxygenation levels in the range 60-100 percent and a constant pulse rate of 70 BPM. Within this range, nine PBV vectors are uniformly sampled (1 = 100%, 9 = 60%). It can be observed from the spectrograms and the PBV indices (red), that the PBV vector corresponding with the pulse signal with the highest SNR, can be mapped to the correct oxygenation level. The PBV indices (red) indicate the PBV vector with the highest SNR for each time-window of 8 seconds.

Up to now, we considered 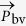 to be static. However, as can be observed from Equation 16, 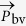 is partly determined by the PPG amplitude spectrum. Different blood oxygen values reflect different mixtures of the absorption spectra of HbO_2_ and Hb, leading to different PPG amplitude spectra. The PPG spectra for 60 and 100 percent SpO_2_ are visualized in Fig. 7. As can be observed from this figure, the PPG amplitude increases for *λ* < 800 nm in the near-infrared part of the light spectrum when SpO_2_ decreases, whereas the opposite holds for *λ* > 800 nm. Consequently, the values of the ‘signature’ 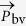 vector change for different blood oxygenation levels. Our method exploits this observation by applying a collection of 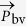 vectors, each corresponding to a specific SpO_2_ value. The values of 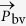 can be determined by Equation 16, where the PPG amplitude spectrum, *PPG(λ*), is the only SpO_2_-dependent term. Since the PPG spectrum is partly determined by a linear mixture of the spectra of oxygenated and deoxygenated haemoglobin, the collection of examined 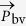 vectors can be expressed as:


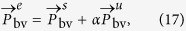


where 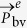 is the examined vector, 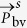 is the static vector corresponding with 100 percent SpO_2_, *α* indicates the gain factor and 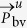 the update vector, which describes the ratio of amplitude changes for decreasing SpO_2_ in the different channels. As can be observed from Equation 16, the values of Equation 17 depend on the selected wavelengths and the optical characteristics of the camera, where the PPG amplitude spectrum can be linearly interpolated within a clinically relevant range of blood oxygenation levels, as visualized in Fig. 7. As elaborated in the previous paragraph, the PBV method calculates the weights 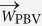 for which the correlation between 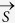 and **C**_N_ equals the blood volume pulse ‘signature’ 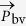. When the relative pulsatile amplitudes of the color channels do not match 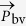, the method will mix in noise to ensure that the correlation between the pulse signal 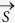 and the normalized color channels **C**_N_ equals 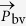 as has been mentioned in ref. 17. Consequently, the quality of the resulting pulse signal reduces when 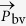 deviates from the correct vector. Since we can calculate 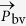 for different blood oxygenation levels, the vector which provides the pulse signal with the highest SNR describes the data best, and can consequently be related back to an SpO_2_ value. An illustrative example of this principle on synthetic data is displayed in Fig. 11.

Essentially, this proposed method inverts the process of measuring SpO_2_ compared to the conventional ratio-of-ratios principle; whereas the ratio-of-ratios principle estimates SpO_2_ from features of the PPG waveforms of the individual wavelengths, our proposed method examines a collection of ‘signatures’ of oxygenation levels, and determines SpO_2_ from the signature which describes data best, based on the quality of the pulse signals. This has the advantage that artifacts present in the PPG waveforms, such as motion and noise, can be eliminated, which is the main cause of erroneousness measurements in conventional pulse-oximetry. Moreover, we are much less dependent on the overall quality of pulse signal because the optimal signature remains stable, even when the pulse signal itself is very noisy. However, a requirement for the method is a correct determination of the pulse-frequency, which is necessary to identify the differences in pulse quality for different 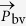 vectors. In the next section we will describe how this “adaptive PBV method” (APBV) is incorporated in the general framework, which additionally exploited the spatial redundancy of the camera, i.e. multi-site measurements robustly combined in a single value, to further improve robustness.

### Framework

**Figure 12:**
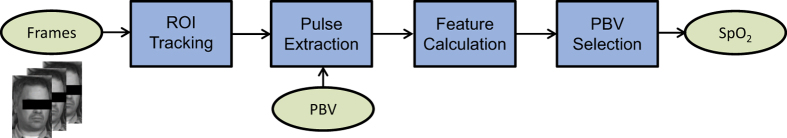
The four operations in the proposed framework. (1) the selected ROI (forehead) is tracked over time and divided into rectangular sub-regions for which the spatial average is calculated (2) the pulse signal is calculated for a collection of PBV vectors each reflecting a oxygen saturation level (3) spatiotemporal features are calculated from the pulse signals to prune distorted regions, and (4) the PBV vector is selected from the pulse signals of the non-pruned regions.

The proposed framework is visualized in Fig. 12 and can be divided into four operations: (1) RoI tracking, (2) pulse extraction, (3) feature calculation, and (4) PBV selection. We will now discuss each operation separately.

- ROI Tracking: The Region of Interest (RoI) comprising skin area is manually initialized in the first frame and tracked over time using the CSK algorithm of Henriques *et al*.^40^, similar to our earlier study^30^. To exploit the spatial redundancy of the camera sensor, the rectangular RoI is divided into *M* equally sized sub-regions for which the spatial average is calculated. By concatenating these values over time for each sub-region and wavelength, traces are constructed, which are consequently used to extract the pulse signals in the next processing step.
- Pulse extraction: The traces of the spatial averages are mean-centered normalized 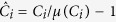 within a time-window of length *L* (default *L* = 150, 10 seconds) to remove the DC component, and are stored in a matrix **C** with dimension *M* × *L* × 3. From these normalized traces, pulse signals are calculated for all *N*_*P*_ examined 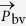 vectors and all *M* sub-regions, and are stored in the matrix **S** of dimension *M* × *L*.
- Feature Calculation: Within the selected RoI, not all sub-regions have similar pulse quality, e.g. they contain non-skin pixels or suffer from local motion distortions. To minimize the effects of these sub-regions in the next, crucial SpO_2_ estimation step, a quality measure for each sub-region is calculated to prune distorted regions. This quality measure, **Q**, consists of two spatiotemporal features: (1) cross-spectral signal-to-noise ratio (SNR), and (2) spectral peak correspondence:

1. Cross-spectral signal-to-noise ratio (SNR): The spectrum of a clean pulse signal consists of peak at the pulse frequency and multiple smaller peaks at the locations of the harmonics. A common metric to express the quality of a signal is the signal-to-noise ratio (SNR), which expresses the ratio between the spectral energy of the fundamental frequency and other components present in the spectrum. Our SNR metric is similar to the one proposed by^37^, and can be expressed as:

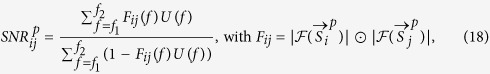

where *U* is a binary template centered around the pulse peak location and its harmonics, ⊙ indicates element-wise multiplication, *i, j* are sub-region indices, and 1 ≤ *p* ≤ *N*_*P*_. The peak location is determined by selecting the peak of the histogram where the histogram is taken over the location of the peak frequency in each of the *N*_*P*_ × *M* pulse signals. Different from^37^, not only the SNRs of each individual sub-region are calculated, but also the SNRs of all sub-region combinations are calculated, closely related to spectral coherence. These cross-spectral SNR values express the spectral correlation of the sub-regions, and are consequently normalized in the range [0,1]: 
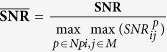
.
2. Spectral peak correspondence: The pulse rate is expected to be similar for all sub-regions within the selected RoI, resulting a similar frequency peaks. Regions with a distorted pulse signal are likely to have a frequency peak different from the pulse frequency. This deviation can be expressed by calculating the spectral peak differences of all sub-region combinations:

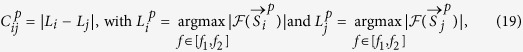


where *f*_1_, *f*_2_ are similar compared to the SNR calculations. The scores 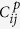 are again normalized in the range [0,1]: 
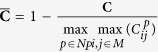
.

**Figure 13:**
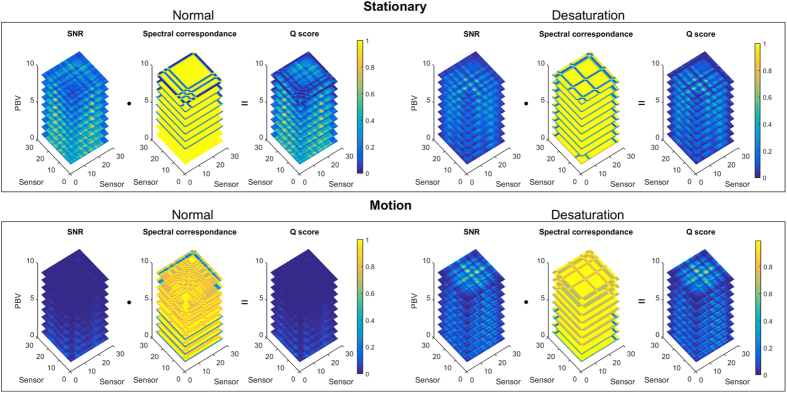
Visualization of the features and quality measure for different scenarios with nine PBV vectors uniformly sampled in the range 60–100 SpO_2_, where PBV 1 corresponds to 100% and PBV 9 to 60% SpO_2_. This quality measure calculated for each sub-region is used to prune sub-regions with low SNR, which could corrupt the SpO_2_ measurements.

The final quality measure **Q** is defined as the element-wise multiplication of the normalized SNR and peak correspondence scores: 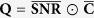. A visualization of the features is displayed in Fig. 13 for different scenarios, with *N*_*P*_ = 9 and *M* = 30.

- PBV Selection: By adding the row-elements of **Q**, a quality measure for each sub-region is obtained, 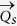, which is used to prune possibly distorted regions: 
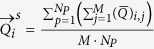
. Sub-regions with a quality measure smaller than 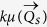 are pruned (default *k* = 1). After pruning the distorted sub-regions, a single pulse signal is calculated for each pulse vector *p* by taking the mean of the pulse traces from the remaining sub-regions. From the *N*_*P*_ pulse signals, the pulse signal with the highest SNR is selected and its corresponding 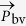 can be related back to an SpO_2_ value, as explained in the description of the APBV method.

Finally, the estimated SpO_2_ values are low-pass filtered using a five-point moving average filter.

### Study

The study has been approved by the Philips Institutional Review Board (Internal Committee on Biomedical Experiments) before experimentation. All experiments were carried out in accordance with the ethical standards laid down in the 1964 Declaration of Helsinki. Informed consent was obtained for each test subject.

## Additional Information

**How to cite this article**: van Gastel, M. *et al*. New principle for measuring arterial blood oxygenation, enabling motion-robust remote monitoring. *Sci. Rep.* **6**, 38609; doi: 10.1038/srep38609 (2016).

**Publisher's note:** Springer Nature remains neutral with regard to jurisdictional claims in published maps and institutional affiliations.

## Figures and Tables

**Table 1:** Statistical results on our benchmark dataset, whereby the sequences are split in a static and motion part.

Subject	A_pulse_	F_pulse_	A_noise_	F_noise_	Static						Motion					
					APBV			RR			APBV			RR		
					MAE	RMSE	STD	MAE	RMSE	STD	MAE	RMSE	STD	MAE	RMSE	STD
I	2.60 · 10^−3^	[51,88]	2.16 · 10^−2^	[12,131]	1.75	2.14	1.55	2.60	2.99	1.84	2.09	2.77	2.47	16.7	18.8	9.50
II	1.11 · 10^−3^	[49,74]	2.83 · 10^−2^	[9,98]	0.72	1.19	1.05	3.72	4.28	3.12	1.66	2.12	1.66	30.2	32.4	14.1
III	2.19 · 10^−3^	[64,87]	1.81 · 10^−2^	[15,107]	0.96	1.16	1.16	1.19	1.56	1.43	1.44	1.85	1.42	21.9	25.5	13.3
IV	2.27 · 10^−3^	[54,109]	3.43 · 10^−2^	[17,114]	1.40	1.73	1.19	2.54	2.83	1.61	2.91	3.60	2.41	27.8	30.5	12.7

For each subject the average pulse and noise (motion) amplitudes are included, together with the range of frequencies of both expressed in beats/movements min^−1^.

**Table 2:** Statistical results of the performance comparison for the wavelength combinations [675,800,840] and [760,800,840] nm on a sequence with motion.

Scenario	[675,800,840]						[760,800,840]					
	APBV			RR			APBV			RR		
	MAE	RMSE	STD	MAE	RMSE	STD	MAE	RMSE	STD	MAE	RMSE	STD
Static	1.17	1.33	0.91	1.62	1.87	1.78	1.21	1.47	1.33	1.69	2.26	2.22
Motion	1.40	1.64	1.44	14.82	15.85	5.61	1.98	2.42	2.33	22.6	24.4	9.15
Average	1.28	1.49	1.17	8.22	8.86	3.69	1.59	1.94	1.83	12.2	13.3	5.69

## References

[b1] Fanaroff A., Fanaroff J. & Klaus M. Klaus and Fanaroff’s Care of the High-Risk Neonate, Expert Consult - Online and Print,6: Klaus and Fanaroff’s Care of the High-Risk Neonate. ClinicalKey 2012 (Elsevier/Saunders, 2012).

[b2] Aoyagi T., Kishi M., Yamaguchi K. & Watanabe S. Improvement of the earpiece oximeter. Japanese Society of Medical Electronics and Biological Engineering 974, 90–91 (1974).

[b3] Allen J. Photoplethysmography and its application in clinical physiological measurement. Physiological Measurement 28, R1–R39 (2007). 17322588 10.1088/0967-3334/28/3/R01

[b4] Eichenfield L. E. & Hardaway C. A. Neonatal dermatology. Current opinion in pediatrics 11, 471–474 (1999). 10555601 10.1097/00008480-199910000-00017

[b5] Wieringa F., Mastik F. & Van der Steen A. Contactless multiple wavelength photoplethysmographic imaging: a first step toward “SpO_2_ camera” technology. Annals of biomedical engineering 33, 1034–1041 (2005). 16133912 10.1007/s10439-005-5763-2

[b6] Humphreys K., Ward T. & Markham C. A CMOS camera-based pulse oximetry imaging system. In *Engineering in Medicine and Biology Society, 2005. IEEE-EMBS 2005. 27th Annual International Conference of the* 3494–3497 (2005). 10.1109/IEMBS.2005.1617232 17280977

[b7] Nishidate I. . Visualizing of skin chromophore concentrations by use of RGB images. Opt. Lett. 33, 2263–2265 (2008). 18830372 10.1364/ol.33.002263

[b8] Scully C. . Physiological parameter monitoring from optical recordings with a mobile phone. Biomedical Engineering, IEEE Transactions on 59, 303–306 (2012). 10.1109/TBME.2011.2163157 PMC3476722 21803676

[b9] Karlen W., Lim J., Ansermino J. M., Dumont G. & Scheffer C. Design challenges for camera oximetry on a mobile phone. In *Engineering in Medicine and Biology Society (EMBC), 2012 Annual International Conference of the IEEE* 2448–2451 (2012). 10.1109/EMBC.2012.6346459 23366420

[b10] Karlen W., Ansermino J. M., Dumont G., Scheffer C. . Detection of the optimal region of interest for camera oximetry. In *Engineering in Medicine and Biology Society (EMBC), 2013 35th Annual International Conference of the IEEE* 2263–2266 (2013). 10.1109/EMBC.2013.6609988 24110175

[b11] Kong L. . Non-contact detection of oxygen saturation based on visible light imaging device using ambient light. Optics express 21, 17464–17471 (2013). 23938616 10.1364/OE.21.017464

[b12] Tarassenko L. . Non-contact video-based vital sign monitoring using ambient light and auto-regressive models. Physiological measurement 35, 807 (2014). 24681430 10.1088/0967-3334/35/5/807

[b13] Guazzi A. R. . Non-contact measurement of oxygen saturation with an RGB camera. Biomedical optics express 6, 3320–3338 (2015). 26417504 10.1364/BOE.6.003320 PMC4574660

[b14] Bal U. Non-contact estimation of heart rate and oxygen saturation using ambient light. Biomedical optics express 6, 86–97 (2015). 25657877 10.1364/BOE.6.000086 PMC4317113

[b15] Liu H., Ivanov K., Wang Y. & Wang L. A novel method based on two cameras for accurate estimation of arterial oxygen saturation. BioMedical Engineering OnLine 14, 52 (2015). 26025439 10.1186/s12938-015-0045-1 PMC4449570

[b16] Shao D. . Noncontact monitoring of blood oxygen saturation using camera and dual-wavelength imaging system. Biomedical Engineering, IEEE Transactions on 63, 1091–1098 (2016). 10.1109/TBME.2015.2481896 26415199

[b17] de Haan G. & van Leest A. Improved motion robustness of remote-PPG by using the blood volume pulse signature. Physiological Measurement 3, 1913–1926 (2014). 10.1088/0967-3334/35/9/1913 25159049

[b18] Mannheimer P. D. The light–tissue interaction of pulse oximetry. Anesthesia & Analgesia 105, S10–S17 (2007). 18048891 10.1213/01.ane.0000269522.84942.54

[b19] Farrell T. J., Patterson M. S. & Wilson B. A diffusion theory model of spatially resolved, steady-state diffuse reflectance for the noninvasive determination of tissue optical properties invivo. Medical Physics 19, 879–888 (1992). 1518476 10.1118/1.596777

[b20] Fine I. The optical origin of the PPG signal. In *Saratov Fall Meeting 2013: Optical Technologies in Biophysics and Medicine XV; and Laser Physics and Photonics XV*, 903103–903103–9 (2014).

[b21] Kamshilin A. . A new look at the essence of the imaging photoplethysmography. Scientific reports 5, 10494 (2015). 25994481 10.1038/srep10494 PMC4440202

[b22] Moço A. V., Stuijk S. & de Haan G. Motion robust PPG-imaging through color channel mapping. Biomedical optics express 7, 1737–1754 (2016). 27231618 10.1364/BOE.7.001737 PMC4871078

[b23] Verkruysse W. . Calibration of contactless pulse oximetry. *Anesthesia & Analgesia (in press* 2016), doi: 10.1213/ANE.0000000000001381. PMC5145250 27258081

[b24] Lamonaca F. . Blood oxygen saturation measurement by smartphone camera. In *Medical Measurements and Applications (MeMeA), 2015 IEEE International Symposium on* 359–364 (2015).

[b25] Particular requirements for basic safety and essential performance of pulse oximeter equipment. Standard, International Organization for Standardization (ISO), Geneva, CH (2011).

[b26] Fitzpatrick T. The validity and practicality of sun-reactive skin types I through VI. Archives of Dermatology 124, 869–871 (1988). 3377516 10.1001/archderm.124.6.869

[b27] Walls R. M. & Murphy M. F. Manual of emergency airway management (Lippincott Williams & Wilkins, 2008).

[b28] Lindholm P., Blogg S. L. & Gennser M. Pulse oximetry to detect hypoxemia during apnea: comparison of finger and ear probes. Aviation, space, and environmental medicine 78, 770–773 (2007). 17760284

[b29] Severinghaus J. W. & Kelleher J. Recent developments in pulse oximetry. Anesthesiology 76, 1018 (1992). 1599088 10.1097/00000542-199206000-00024

[b30] van Gastel M., Stuijk S. & de Haan G. Motion robust remote-PPG in infrared. Biomedical Engineering, IEEE Transactions on 62, 1425–1433 (2015). 10.1109/TBME.2015.2390261 25585411

[b31] Bickler P. E., Feiner J. R. & Severinghaus J. W. Effects of skin pigmentation on pulse oximeter accuracy at low saturation. Anesthesiology 102, 715–719 (2005). 15791098 10.1097/00000542-200504000-00004

[b32] Kanzawa Y., Naito T. & Kimura Y. Human skin detection by visible and near-infrared imaging. *IAPR Conference on Machine Vision Applications* **12**, 14–22 (2011).

[b33] Mendelson Y. & Ochs B. D. Noninvasive pulse oximetry utilizing skin reflectance photoplethysmography. Biomedical Engineering, IEEE Transactions on 35, 798–805 (1988). 10.1109/10.7286 3192229

[b34] Mendelson Y. Pulse oximetry: theory and applications for noninvasive monitoring. Clinical chemistry 38, 1601–1607 (1992). 1525987

[b35] Wukitsch M. W., Petterson M. M. T., Tobler D. R. & Pologe J. A. Pulse oximetry: analysis of theory, technology, and practice. Journal of Clinical Monitoring 4, 290–301 (1988). 3057122 10.1007/BF01617328

[b36] Poh M.-Z., McDuff D. J. & Picard R. W. Advancements in noncontact, multiparameter physiological measurements using a webcam. Biomedical Engineering, IEEE Transactions on 58, 7–11 (2011). 10.1109/TBME.2010.2086456 20952328

[b37] de Haan G. & Jeanne V. Robust pulse-rate from chrominance-based rPPG. IEEE Trans. on Biomedical Engineering 60, 2878–2886 (2013). 10.1109/TBME.2013.2266196 23744659

[b38] Huelsbusch M. & Blazek V. Contactless mapping of rhythmical phenomena in tissue perfusion using PPGI. Proc. SPIE 4683, 110–117 (2002).

[b39] Corral Martinez L. F., Paez G. & Strojnik M. Optimal wavelength selection for noncontact reflection photoplethysmography. 22nd Congress of the Int. Commission for Optics 8011, 801191–7 (2011).

[b40] Henriques J. F., Caseiro R., Martins P. & Batista J. Exploiting the circulant structure of tracking-by-detection with kernels. In *European Conference on Computer Vision (ECCV) 2012*, 702–715 (2012).

[b41] Prahl S. Tabulated molar extinction coefficient for hemoglobin in water. http://omlc.org/spectra/hemoglobin/ (1998) (accessed: 10/03/2016).

